# Are young adults appreciating the health promotion messages on diet and exercise?

**DOI:** 10.1007/s10389-018-0905-9

**Published:** 2018-02-19

**Authors:** Emma Berry, Lorna Aucott, Amudha Poobalan

**Affiliations:** University of Aberdeen, Foresterhill, Aberdeen, AB25 2ZD UK

**Keywords:** Young adults, Diet, Exercise, Health promotion

## Abstract

**Aim:**

This study aims to determine if current health promotion messages relating to diet and physical activity are sufficiently targeted towards young adults. In addition, we examine what elements of these messages might be improved to ensure they encourage improved diet and exercise behaviours within this underserved group.

**Subject and methods:**

Using qualitative methods, five focus group discussions (FGDs) and two semi-structured in-depth interviews were conducted among 19 young adults in Aberdeen City. An appropriate topic guide was developed for this purpose. After obtaining consent, all FGDs and interviews were audio-recorded and transcribed verbatim. A thematic analysis was conducted that allowed for emerging themes to be identified from the data. Links between themes were established and key quotes identified.

**Results:**

Five major themes emerged: (1) exposure to health messages over time; (2) chains of healthy or unhealthy behaviours; (3) perceptions and attitudes towards health messages; (4) facilitators and barriers; (5) improving the usability of health messages.

**Conclusions:**

The results demonstrate that young adults did not find current health promotion messages engaging. These messages did not support them in overcoming their perceived barriers, nor were they suitably formatted or located for them. There were suggestions from young adults on how to improve these messages including using social media, presenting messages in more usable forms, and working with larger corporations to make these messages more effective. Tailoring these messages specifically for young adults could improve their diet and exercise behaviours, thereby helping to reduce future obesity levels and co-morbidities within Scotland.

**Electronic supplementary material:**

The online version of this article (10.1007/s10389-018-0905-9) contains supplementary material, which is available to authorized users.

## Introduction

Obesity is a major public health problem in Scotland, with the Scottish Health Survey from 2015 showing 65% of adults classified as overweight, of which 29% were obese (Scottish Government 2016). Obesity increases many disease risks including type 2 diabetes and cardiovascular disease (Seidell and Halberstadt 2015). The cost of obesity and obesity-related diseases to the National Health Service (NHS) is staggering, suggested to be £2.47 billion (Tovey 2017). The cost and health complications arising from obesity make it an important problem to tackle, both quickly and effectively.

Young adults have been shown to have the quickest rate of weight gain out of all the age groups (Ng et al. 2014). This age is critical as obesity within this age group continues into adult years and increases the risks of chronic diseases later in life (Gordon-Larsen et al. 2010). Young adults undergo various life course changes such as leaving home and starting university or full-time work. For those starting university life, research has identified that finances, social pressures, and time are all barriers to healthy choices (Nikolaou et al. 2015), but unfortunately young adults who do not continue onto further education are poorly recruited for health research (Poobalan et al. 2016). Nonetheless, it is important that weight management efforts also target young adults in all of their environments. Despite their vulnerability, this age group presents possible teachable moments for public health strategies that might encourage healthy behaviours now and for future health (Giles and Brennan 2015).

One of the most common ways to improve health is through communicating health promotion messages, with targeted messages being shown to be the most effective in altering behaviours (Green et al. 2015). Current strategies in Scotland to improve obesity rates are aimed at young children and families (Scottish Government 2010). This may improve obesity levels in the future, but it does not tackle the young adult age group where weight gain is the most rapid (Ng et al. 2014). Baker et al. (2010) demonstrated that targeted health promotion messages can improve healthy eating in young adults. Despite this, targeted messages for this age group on diet and exercise in the community are lacking as we found in our previous literature review (Berry et al. 2017). Young adults are in a time of transition and the current health messages (HMs) are not suitable to encourage healthier behaviours in diet and exercise for this group (Giles and Brennan 2015).

Recent evidence demonstrates young adults are aware of HMs surrounding diet and exercise but are not sufficiently motivated by them to improve behaviours (Giles and Brennan 2015; Poobalan et al. 2012). Giles and Brennan (2015) found that young adults are aware of HMs on exercise and diet but found these to be unrelatable, mainly because of messages targeting families or older adults. Although this identifies that the current health promotion messages do not engage young adults (Giles and Brennan 2015), there is limited research on how HMs could be improved to better target them and their life situations. Therefore, the aims of this study are to assess the perceptions about health promotion messages on diet and exercise for young adults and determine how they might be improved for this age group.

## Design and method

### Study design

This study was carried out to establish young adults’ awareness of current HMs in diet and exercise and their perceptions of these messages and to explore their suggested improvements. A qualitative approach was used to gather in-depth understanding of young adults’ perceptions and opinions on this topic. Focus group discussions (FDGs) were deemed suitable for exploring this topic, as this would promote a dynamic interaction in this age group. However, when it was not possible to get a group together, individual interviews were conducted using the same topic guide, so as to not lose the valuable information from this hard-to-reach group. Although different methods were used to collect data, they were subject to the same rigorous method of analysis (Ritchie et al. 2014).

### Participants

Young adults aged between 18 to 25 years were recruited through convenience sampling within the City of Aberdeen and attended sessions during May and June 2017. Recruitment aimed for a diverse sample, with respect to socio-economic status (SES), gender, and educational background. Participants were recruited using flyer advertisements and contacting leaders of pre-formed groups of young adults throughout the city. We aimed to recruit from a variety of socio-economic backgrounds—judged by location. Focus groups consisted of participants in pre-formed groups or relationships, including pre-existing friend groups or social clubs. Interviews were used when numbers were too small for a focus group. Interviews and focus groups were conducted in participants’ natural settings, during times that suited the individual/group.

### Data collection

Participants gave informed consent before taking part. Both the FGDs and semi-structured in-depth interviews were based on the topic guide, specifically developed for the purpose of this research (Supplementary material 1). These questions were informed by a previous literature review (Berry et al. 2017), carried out before the study commenced. While conducting FDGs and interviews, health promotion materials that were readily available in Aberdeen at the time were collected and used as prompts to facilitate the discussions. All sessions were conducted by EB as the main interviewer. Discussions were recorded using an audio recorder.

### Data analysis

All recordings were transcribed verbatim and anonymised. Thematic analysis was used to analyse the data. Initially, all the transcripts were read several times to familiarise with the data. Following this, recurrent data that had similar patterns were coded. An initial list of the items was discussed with AP and grouped into categories and evolving themes were identified. Emerging themes from each transcription and corresponding quotes were noted. Links between themes were established to identify key areas of how these health promotion messages could be improved for young adults. Data were collected until no new key themes emerged. At this point, one further focus group was conducted to ensure that data saturation was reached.

## Results

### Participant demographics

Nineteen participants were recruited forming five focus groups and two interviews. The characteristics of the participants are shown in Table 1. Once consented, participants completed a short demographic questionnaire, determining any potential influencing factors (Table 2).

**Table 1:** Table summarising the characteristics of each session

Session number	Code	Characteristics	Number of participants	Age range (years)	Female: male	Working: studying
FG1	B1-4	University students in organised sports club	4	19–22	1:3	0:4
FG2	N1-6	Nutrition university students	6	21–25	6:0	0:6
FG3	C1-3	Undergraduate university students—1 recently finished, others mid-way through courses	3	18–22	2:1	1:2
FG4	R1-2	Mixed—student in sports science and psychology and working indivdual	2	18	0:2	1:1
FG5	F1-2	Full-time working individuals	2	23–25	0:2	2:0
I1	P1	NEET	1	22	1:0	1:0
I2	S1	College student in media	1	19	1:0	0:1

*FG* focus group discussion, *I* semi-structured interview

**Table 2:** Summary of participant demographics split by gender

Participants' demographics summary (*n* = 19)		
Gender (number of participants)	Female (*n* = 11)	Male (*n* = 8)
Mean age (years)	22.18	20.4
Percentage currently studying	72.7%	62.5%
Education level previously achieved	University education: 72.7% College education: 9.1% School qualifications: 18.2%	University education: 12.5% College education: 37.5% School qualifications: 50%
Individuals with further education aspirations	81.8%	100%
Number of exercise sessions per week (30 min or more)	1–2/week: 9.1% 3–4/week: 54.5% 5+/week: 27.3%	1–2/week: 25%% 3–4/week: 37.5% 5+/week: 37.5%
Participants who play a sport	27.3%	87.5%
Time spent sitting per day (hours)	4 or less: 18.2% 5–8: 27.3% 9 or more: 45.5% Other: 9.1%	4 or less: 50% % 5–8: 25% 9 or more: 12.5% Other: 0%
Participants expressing interest in healthy foods	100%	100%
Current living status	On own: 36.4% Friends: 27.3% Family: 27.3% Other: 9.1%	On own: 25% Friends: 37.5% Family: 37.5% Other: 0%

### Key emergent themes

Five major themes emerged during the analysis: (1) exposure to health messages over time; (2) chains of healthy or unhealthy behaviours; (3) perceptions and attitudes towards health messages; (4) facilitators and barriers; (5) improving the usability of health messages. Supplementary material 2 demonstrates how, within one transcript, these themes emerged after several iterations. What now follows is a description of each theme in more detail with illustrative quotes from participants [labelled with their participant code (Table 1) and gender (m/f), e.g. C1f is a female university undergraduate].

#### Exposure to health messages over time

Health knowledge arose from different life stages, discussed as learned behaviours and messages from school, childhood, and family. These were consistent regardless of the background:

*“…when we were in school we used to eat rubbish all the time at break times and to give us a better idea of what we should be eating. I think it was one of the classes to try to get us to stop eating crisps so much”.* (P1f)

Some participants were aware of this information but did not feel it improved their knowledge:

*“Yeah it’s always in classrooms but they never actually talk about it”.* (B3m)

Health knowledge and behaviours in individuals’ current environment, at work, college, or university, was discussed. There was an awareness of HMs, but health knowledge or support for healthy behaviours was felt to be inadequate. Individuals in employment were more aware of HMs in their offices compared to participants undertaking further education:

*“…I see that in my work…They normally just have it in canteens and if you go for your coffee breaks they have it there…every time you have a coffee, make sure you have a glass of water. They were quite good at keeping on at you for that”.* (R1m)

#### Chain of healthy and unhealthy behaviours

Behaviours were expressed by participants as healthy or unhealthy, forming a self-perpetuating chain:

*“Because once you’ve started to be healthy…you start off really gradual and slow but once you get the hang of it you just want to stick, to continue on and stay to be healthy. You just have that motivation, that drive to be more healthy…*”. (C1m)

Behaviours could be perpetuated or interrupted through different external influences. Factors that interrupted a healthy cycle or promoted an unhealthy cycle could be alcohol, social activities, stress, or boredom.

*“Yeah, it gets boring and that’s why I eat other stuff and not healthy stuff*”. (P1f)

However, whether the response to interruptions was healthy or unhealthy depended on the individual and did not differ with gender:

“…*I would feel really stressed so I actually go out and jog around…you feel less stressed as well so…exercise is a really good substitute when you’re revising for exams*”. (C1m)

“*Or you can just eat your sorrows away*”. (C2f)

Participants discussed influential sources they deemed reliable. Sources of trust included trustworthy figures, role models, observable social norms, and other individuals they might relate to. These could motivate individuals into undertaking either healthy or unhealthy behaviours—they tended to copy the behaviour observed:

*“Yeah, The Rock, Hugh Jackman, people like that, they are big. Like Zac Efron, Baywatch is ripped! There are so many people that have went out, he did not look like that in ‘High School Musical’—what do you do to get to that point?”* (F2m)

“*Do you remember like, a few months ago when like the 'Dad Bod' started kicking off…and like within…like male culture it was…like promoting an unhealthy way of living was actually seen as socially quite popular…”*. (B4m).

There were contradictions about what were believed to be trustworthy sources of health information:

*“…I don’t necessarily go on the NHS or anything. I would go for* *Bodybuilding.com**, Men’s Health, that kind of stuff…*”. (R1m)

*“…if it’s not a website that I’ve known of or not NHS or something in government then you’re a bit wary because anybody can just write anything they wanted*”. (P1f).

Fear and/or stigmatisation could prevent individuals undertaking healthy behaviour:

“*If you see an overweight person in the gym, there is always the stereotype of guys who want to laugh at you…*”. (F2m)

However, realisation of negative consequences from unhealthy choices could motivate an individual to be healthier:

“*…when I started to see changes in myself and when I wasn’t feeling as well, that’s when I really started thinking about reaching out for my fitness pal and stuff like that*”. (C3f)

#### Perceptions and attitudes towards health messages

Participants discussed their perceptions and attitudes towards HMs. There was a perception of an abundance of unhealthy messages and few healthy ones:

“*…it feels like every time I walk down the street I see messages for fast foods. I don’t really see a lot of things standing out for health, and all that, so it feels like you are just bombarded with unhealthy messages, rather than healthy messages. I guess, it’s kind of discouraging". *(S1f)

Participants discussed such messages; they could be passively observed, or the individual might actively look for them. Most were observed using the internet:

*“I see them on billboards, maybe if you are in the car, or if you are walking, or on the bus, you see them going past for fast food, restaurants, even on TV…Also, when you are on your phone and when you are scrolling, like adverts pop up messages…They have adverts, it’s at the start of videos and they are very annoying”.* (S1f)

“*You know you’ve got that men’s health Facebook page that I follow*”. (B2m)

Young adults discussed the role of large corporations, suggesting that they did not care about customer health, which could indirectly encourage young adults to be unhealthy:

*“…like advertisement agencies don’t really discriminate between 'oh this is a more important message than drinking coke*'”. (B3m)

#### Facilitators and barriers

Participants discussed factors they perceived to be barriers to healthy choices. Observed barriers included convenience, rigidity of messages available, and accessibility:

“*But the thing with it is that leaflets like that never say anything about you can eat this, just in small quantities or just on an irregular basis, just every now and again. They need to express that it’s okay to eat that now and again…you have to treat yourself now and again”.* (R1m)

They felt that the HMs available did not reflect a suitable range of goals and targets for young adults, thus creating barriers. However, male participants discussed this more than the females:

*“So…(R1m), you’re going for lean, I’m trying to put on weight and you’re changing your muscle mass. It’s all very specific in books and posters and leaflets, it’s usually aimed at people losing weight from fat to skinny”*. (R2m)

Although clear barriers were discussed, many factors were viewed as being both facilitators and barriers depending on the individual. Such factors included time, cost, setting targets or goals, and competition. One example, ‘time’, was perceived as a barrier, but targeting time constraints could facilitate healthier behaviours:

“*One thing is also finding the time cause like when I was working before I went to uni…I didn’t really have that much time to actually do any exercise. The only reason I am actually quite fit at uni is because I have buckets of time to go for runs and site train, watch what I’m eating but for like young adults who actually have a job it’s like really difficult”.* (B4m)

*“…you’ve got like app at home like the 7 minute app or something, I feel like that helps as well cause its only 7 minutes…I know people do it because it’s…such a short time and it’s not like long like a three-hour long gym session and it feels more like achievable I guess".* (B1f)

The cost of making healthier choices was a barrier. However, reference to the internet and social media for health advice, free resources, was demonstrated as a facilitator:

*“You don’t want to go and spend £12 or £13 on a book that’s going to tell you how to cook a meal when you can just Google it”*. (R2m)

Participants discussed setting their own short-term goals to achieve healthier lifestyles. Such goals helped by breaking things into achievable tasks or short-term outcomes. However, if not achieved these could turn into barriers:

*“I think it’s just like the little achievable tasks…like really small but you do achieve it and you feel more motivated than if you like said like I’ve just planned out my week…to run three times, got to eat this many meals like if you even start like not achieving the smallest tasks…you get so demotivated and then just almost like again it snowballs with that you just like don’t do any of it*". (B4m)

Participants discussed how food appearance and taste facilitated healthy choices but equally promoted unhealthy foods, again depending on the person:

“*It’s probably like, companies make their food look nicer, so it appeals to more people…I’ve never seen a fruit and vegetable company saying, 'Look at our crisp juicy apples. Mmm'…They make food look a lot better that it really does when it comes out in the store. Just entices people to come in, more people to come in and eat”*. (S1f)

The impact of competition and challenges was discussed with some being motivated into healthy behaviours:

*“But I do challenges with my grandma because she’s retired so I’m busy trying to keep up with her”*. (P1f)

Others expressed no interest in competition and might even be demotivated by it.

*“I’m the opposite. I’m not very competitive”*. (F1m)

#### Improving the usability of health messages

Participants discussed different resources available for HMs to be viewed and the formats in which it was presented. These included videos, apps, leaflets, posters, and social media posts:

“…*its either in like just picture style or like video style cause like I know people are lazy with…reading. I’m one of them*…”. (B2m)

*“I know on a lot of social media platforms now there’s sponsored posts, so as you’re scrolling down one will come up…I have been occasionally stopped being like, 'Oh, what is that?'”* (N3f)

Participants discussed the messages and campaigns currently available and their perceptions of these:

*“…The campaign I’m really aware of is This Girl Can because they have amazing TV adverts, amazing internet, their Twitter is really good so I think it just depends how much effort is put into the promotion of that health message”*. (C3f)

Improvements for these messages were discussed. Improvements in presentation to increase memorability included eye-catching posters or theme tunes, making them more noticeable, memorable, and catchy:

*“I think especially for students, posters are more effective because no-one would like to read a long paragraph about healthy eating so maybe if there’s a strong graphic or a slogan…*”. (C1m)

Other improvements suggested were to make messages that targeted perceived barriers, linking messages with usable forms, using social media for promoting health and using behaviour-specific reminders to encourage young adults to be healthier.

*“…the ones that said, 'Try this for ten days and see the difference' and when you do and you’re like, 'Oh, this is actually really good' and you continue on…”*. (C2f)

*“I usually go to like BBC GoodFood…Cause they’ve got really nice recipes…it like explains it as well like why it’s healthy sometimes…and what you should do if you’re following this particular lifestyle or something”*. (B1??)

*“…I saw something on the news a little while ago about how Instagram has helped so many people improve their eating. I know obviously there’s lots of negatives to that, but there are groups where everybody posts everything they eat in a day and that can really help people to be inspired and be, 'Oh, they’re eating a beautiful colourful plate of food, that’s what I want to do too'. So I don’t know, maybe for our age group at least these traditional messages may be lost, but there are other ways I guess”*. (N5f)

“*If there was something specific, I would go…and download an app to remind me if it’s water-related…to get notifications…”.* (C2f)

Although participants felt that larger corporations often promote unhealthy behaviours, they also noted that some used positive apparently personalised messages and felt that working with these corporations would be beneficial:

"*I think if they…worked with big companies as well like Tesco’s or Co-op…like Tesco’s has like on…the side of busses…advertising 'this is Sophie’s paella' or whatever–As like encouraging*". (B2m)

## Discussion

This study confirmed that HMs about diet and exercise for young adults need to be targeted to them specifically. It provides deeper, richer information about the knowledge, perception, and awareness of health and HMs among young adults from a spectrum of backgrounds. To our knowledge, this is the first study investigating how messages could be made more effective and appropriate for young adults rather than just describing the influencing factors. Five distinct themes emerged, containing ideas in line with recent research, but also others with further constructs and recommendations for consideration.

The Scottish Government (2010) have targeted young children and families to reduce obesity levels. Our study shows that young adults are aware of these messages received from a young age. However, evidence supporting the translation of awareness into healthier behaviours is mixed. Some suggest that education-based interventions on healthier choices in children do not improve long-term behaviours (Griffin et al. 2015), although incentives may make these more effective (Loewenstein et al. 2016). While targeting children is well evidenced, our young adults struggled to identify with many of the HMs they received, often dismissing them as not being relevant now. Some individuals had been exposed to current HMs that reinforced previous ones but our study indicates that this varied depending on their current environment. This was the only difference observed between the group responses based on their backgrounds and current environments: Young adults in further education claim these messages are missing, whereas working individuals could recall HMs within their workplace. These differences are possibly due to the Scottish Healthy Working Lives initiative, which has seen a substantial increase in health promotion activities within the work environment (NHS Health Scotland 2012). However, many in this age group will not be employed, and it is important that all have access to appropriate health promotion material regardless of their environment. There is evidence to suggest that targeted messages within further education settings can be effective (Baker et al. 2010).

The young adults in this study discussed healthy and unhealthy behaviours as chains of self-perpetuating action. The development of these behaviours is complex. Autonomous motivation, self-efficacy, and self-monitoring have all been shown to increase an individual’s likelihood of undertaking health behaviours (Teixeria et al. 2015). Unhealthy behaviour chains could result from a decrease in these factors by people *“giving up”*. However, behaviour chains may be interrupted by external influences, e.g. social activities and examinations. Unfortunately, reactions to these influencers often depend on the individual and their previous habits. This would explain the different reactions of individuals to the same external influence——some being healthy or unhealthy (Gardner 2015). This has been consistently reported by young adults who find that when phases of healthy behaviour break, they often fall back into bad habits (Poobalan et al. 2014). This was a recognised problem for the young adults in this study, but our young adults went on to suggest that a solution might be for health promotion messages to also support individuals in countering the bad habits in order to be more successful in achieving the healthier ones.

For this age group, behaviour change depends on trust. Our young adults discussed how figures of trust, social norms, role models, and other relatable individuals can all influence their choices. Sbaffi and Rowley (2017) also discussed factors influencing an individuals’ trust in health information including the source of expertise and the individual’s intention to use the information. The main source of the material tends to be social media and the internet for this age group (Sbaffi and Rowley 2017). In our study, trust in specific sources varied between participants and was often contrasting. This could be due to differences in opinions about what makes a trustworthy source, the health goals individual’s set, and how they plan to use the information. Messages therefore should be conveyed through a trustworthy or endorsed source as determined by the recipients at the time and so may change.

We found that fear influenced behaviour. Participants discussed the fear of being ridiculed and stigmatised; for example, a large person could be laughed at in the gym. The fear of weight stigma has been well documented, with evidence suggesting that it manifests itself in individuals with a self-perception of being overweight and can encourage further unhealthy choices and reduced self-efficacy for self-control (Major et al. 2014). Overall, this may result in unhealthy chains of behaviour. However, fear may also motivate change toward healthy choices. Fear of consequences is often used to motivate individuals within health promotional materials (Ruiter et al. 2014). Our participants recognised that there were consequences of their health behaviours and thus their susceptibility. However, fear as a motivator is only effective when an individual’s self-efficacy is sufficient for them to change their behaviour. Awareness of personal susceptibility for these consequences often increases the likelihood of fear being motivational (Ruiter et al. 2014). Therefore, using fear to motivate health is likely to be limited in participants with poorer self-efficacy and health awareness (Ruiter et al. 2014). In our group, many of the consequences that they thought would make them change their behaviour were image-based or concerned with their current well-being rather than any longer-term health fears such as cardiovascular disease. This was also observed in other studies (Poobalan et al. 2012), suggesting that messages should focus on more immediate consequences rather than longer-term health fears as can happen with older age groups and/or those with specific co-morbidities.

Our young adults felt bombarded by unhealthy message advertisements and observed few healthy ones. They suggested that large corporations were interested in profits rather than customer health. Giles and Brennan (2015) mirrored these findings; their young adults perceived more unhealthy food advertisements compared with healthier messages, making it harder to resist these foods. In our group, messages were either passively or actively observed via various means, again with the internet featuring heavily. Messages actively looked for were often interlinked with trusted sites or sources as discussed previously. Individuals could actively “*follow*” pages giving health advice they believed to be trustworthy, but they also described passively observing messages either online or in their everyday life in the form of adverts. If too frequent, these messages were classed as “*annoying*” and could result in negative consequences regardless of the message. Vaterlaus et al. (2015) also found this—in our study the young adults further describe individuals who frequently posted as trying to show-off. Thus, messages targeting young adults should be appropriate in terms of volume and location to be engaging.

Factors influencing health as barriers or facilitators were often viewed differently in this study depending on the individual. Some were clear barriers such as convenience, accessibility, a lack of variability in messages, and individual targets not being reflected. Young males in our groups classed the limited range of messages to be a barrier more than females did; for instance, they were more interested in becoming muscular than in getting messages about losing weight. Such goal differences have been described in other studies with adolescents (Strandbau and Kvalem 2012); along with the risk of eating disorders in this age group (Loth et al. 2014), messages probably should focus on being healthy rather than totally on weight loss and so instead promote a realistic approach to healthy lifestyles. Our participants also discussed the rigidity of current messages, describing them as having an all-or-nothing approach; they could not recall any messages encouraging an occasional treat as part of a healthy diet.

In terms of facilitators, this study found there to be no one factor. Instead, factors were a facilitator for some but a barrier for others depending on the individual and their circumstances. This is contrary to other studies where stress, time, accessibility, and cost have been described as clear barriers (Nikolaou et al. 2015) and setting goals or targets and healthy peers as consistent facilitators (Nikolaou et al. 2015). While our participants believed accessibility was a barrier, some thought time and money constraints could actually be motivational. A lack of time could encourage shorter bursts of exercise, and a lack of money made them more ingenious using social media or the internet to find free health advice. Goal setting was believed to be motivational (Nikolaou et al. 2015), but our participants suggested that was only the case when goals were achievable so as not to have a negative effect. Competition and challenges deemed motivational in previous research (Nikolaou et al. 2015) we found to be conditional on the individual having a competitive nature and required something or someone to compete against. The effect of food appearance and taste has mixed reviews (Nikolaou et al. 2015; Giles and Brennan 2015), some stating that healthy food's appearance and taste are motivational, with others finding these foods to be “*boring*”. Our results again suggest there is an individual preference, some thinking that social media posts could encourage young adults to view healthy foods as attractive. Social media has been found to promote healthy eating with young adults and to increase the accessibility of healthy foods for this age group through access to free recipes and healthy food pictures (Vaterlaus et al. 2015). As a consequence of our findings, facilitators to encourage healthy behaviours should not be generic with young adults but personalised dependent on their environment and resources available. Health messages should target young adults perhaps using social media (or the most popular platform of the time); they should focus not only on facilitators, which vary from person to person, but should also include tips on how to counter a range of barriers or trigger points.

Our young adults indicated the need for improvements with respect to the formats and contexts of messages. They preferred images and videos over text alone, in line with other studies (Soetens et al. 2014). Social media and the internet unsurprisingly feature heavily as preferred platforms to improve usability, given that young adults are the highest users of social media (Perrin 2015). However, our young adults also proposed working with larger corporations to promote healthy messages as a way of engaging their age group, a suggestion supported by Giles and Brennan (2015). In terms of how to present health promotion messages, our young adults thought that more practical forms such as recipes could be beneficial, and behaviour-specific reminders might aid behaviour change, a point also concluded by Brown et al. (2014).

### Strengths and limitations

Our study confirmed that young adults need to be treated differently and also successfully determined aspects of health promotion messages that could be improved on for their age group. Recruitment through pre-existing groups was successful and encouraged richer data during the sessions. However, organising larger groups proved difficult. Our study had an even gender spread, but poor representation of unemployed participants and socio-economic diversity, which at recruitment had to be determined by the session site location. Even our demographic questionnaire could not accurately define individual SES. Traditional methods of capturing SES based on where a person lives (Shavers 2007) are often inappropriate for young adults, who often live in a temporary accommodation, which may not reflect their true SES. This presents challenges for future research and here restricts the possibility of defining SES groups or persons. While the number of those in employment was small (*n* = 4), this in fact is probably a fair reflection of the community who are of an age where some sort of further education is very much encouraged by government policy.

Participants involved were self-selecting and expressed an interest in healthy foods or exercise through the demographics questionnaire, suggesting they already had an interest in health. As a result, it is possible our young adults were aware of the researchers’ interest in health and reflected this as their own. Either way, these participants may not fully represent this age group and hence bias the responses.

## Conclusions

Our study demonstrates that health messages about diet and exercise are not engaging young adults. In consultation with young adults, messages could be improved with respect to content, presentation, delivery format, and location. Ideas such as using social media, presenting messages in usable forms like recipes using colourful images and using behaviour-specific reminders were all ideas our young adults felt would appeal to their peer group. Young adults within the working environment were more aware of HMs, but face similar barriers as individuals within further education. Therefore, targeting more further education facilities with specific health messages for young adults is important. However, it is also important to engage young adults not in further education or employment. The involvement of larger corporations may counter the unhealthy messages observed and overcome the barriers discussed. Social media/internet platforms could improve access, but messages must be in preferred formats with trustworthy organisations or individuals communicating them.

Overall, messages need to be easy for young adults to act on to encourage behaviour change and should also provide options for when good behaviours break down. The difference in environment and resources available between young adults meant that the facilitators to healthy choices were not consistent and consequently a personalised approach may be required. Messages should be about being healthy rather than focussing on weight loss, to encourage a balanced lifestyle that may include occasional treats, thus reflecting real life. It was also apparent that these messages should focus on immediate consequences from healthy choices rather than long-term health fears. The wider environments need improving by limiting advertisements and access to unhealthy foods, especially within working and further education environments. Further research still needs to illicit further views of those not in further education or employment. There also needs to be an ongoing consultation to determine which sources young adults find trustworthy. These changes and considerations could improve the health of young adults and help to reduce obesity and other co-morbidity rates seen within Scotland.

## Electronic supplementary material

ESM 1 (PDF 546 kb)
