# Supplementary material for: Are young adults appreciating the health promotion messages on diet and exercise?
Source: Z Gesundh Wiss. 2018 Feb 19;26(6):687–96. doi: 10.1007/s10389-018-0905-9 (PMC6245086; doi:10.1007/s10389-018-0905-9)
Supplement: Supplementary file 1 — (PDF 546 kb) [file 10389_2018_905_MOESM1_ESM.pdf]

Title: Are young adults appreciating the health promotion messages on diet and exercise?

Authors: Emma Berry (MSc Student, University of Aberdeen), Dr Lorna Aucott (University of Aberdeen) & Dr Amudha Poobalan (University of Aberdeen)

Author's Addresses:

Health Services Research Building

Forresterhill

Aberdeen

AB252ZD

Author for correspondence:

Dr Lorna Aucott,

Email: l.aucott@abdn.ac.uk

Telephone: 01224 438160

Fax: 01224 438165

Supplementary Materials

Table S1: Pre-determined focus group question list for use during discussion.

Pre-Determined Participant Questions for Focus Groups
**1) What health messages about diet and physical activity have stood out to you?**
**2) Where did you see them?**
**3) What do you think about these health campaign messages?**
**4) What changes, if any, would you make to these campaign messages if you could?**
**5) In general, where would you look for help on healthy eating or physical activity?**
**6) Can you tell me a bit more about why you look there for this information?**
**7) When do you look for information on healthy eating and physical activity?**
**8) Is there anything else you think is important about health messages in diet and exercise that we haven’t covered already?**

Table S2 –Further quotes categorised into themes and sub-themes discussed in the paper

Theme	Sub-theme	Quotes
**Exposure to Health Messages Over Time**	Health knowledge and behaviours from life stages.	*"when we were still at school… you know you're never taught how to cook…"* (B4m)
		"I started eating like you know chia seeds and stuff... cause my parents told me it was good for me." (B2m)
		"I think it's just from my home cause… we've always had vegetables and um potatoes next to it and that so like a piece of meat as well…" (B1f)
	Previous message experience and effect on health knowledge.	*"You get it all from the school at a low level and it crops up occasionally around your everyday life. It's not exactly exhaustive"* (R2m)
	Health message awareness in current environment.	"*You'd think the uni would have more like brochures and stuff."* (N2f)
		"...I've not seen anything in college to do with any of this." (S1f)
**Chain of Healthy and Unhealthy Behaviours**	Behaviour as a chain	"*No you just don't have training for like another three months and I've just given up, not given up... but yeah given up.*" (B4m)
	Factors which influence behaviour chain.	*"I think my diet recently has been quite bad cause I have been going out quite a lot, so I've just not eaten anything all day and then at night I'll be like aw I've got nothing to eat*" (B2m)
		"Yeah, you have to just eat whatever they're eating and you can't say, "No, I'm not eating that because it's not healthy"."(P1f)
		"…I usually run at night but then like… something else comes up at night… I drop running and like go do something else." (B1f)
	Differences in individual actions with same influencing factor.	*"I like to wait until later in the day you know I've had my breakfast you know I've like woken up and I'm like ok I'm not doing anything right now… there's nothing really good to watch I may just as well go out… for a run and freshen up a little bit or if I'm like studying you know if you go out for a run you said earlier it makes your brain feel clearer… you know. That's why I would do it.* " (B2m)
	Figures of trust, role models, social norms and relatability on influencing behaviour.	"*Well every other celebrity you know, you've got Katie Price has brought out now shakes, so you know."*(N1f)
		"*Especially with girls, always aiming for these stupid TV shows and these reality shows and they inspire to be things that are unrealistic. But the same with guys that are trying to be a big ripped guy where it's unrealistic, it's not healthy*."(R2m)

		"*It's a 12-week course and it's a guy from America … he got himself in a state where he was really out of shape and in the 12 weeks he did a video showing every day him making progress and proved that that 12 weeks, if you did it by the book all he was saying, anyone can do it… That's the kind of thing that hard hits me, seeing someone do it and he's doing it to try to get people to do it as well, he's not just doing it for himself.* " (R1m)
		"*Just a lot of other people losing weight and stuff, I think that promotes you to try and eat healthy as well. With **** and a lot of other people I know that are Slimming World and they're always posting pictures of what they're eating on Instagram, it's quite good. A lot of them put on what they're eating and then if you message them they'll tell you how to make it.*"(P1f)
	Contradictions seen in sources of trustworthy information	"*I would definitely check my instructor's web page, like I have a yoga instructor and she gives advice on that too. Maybe it's not scientific but I trust her advice…*" (N6f)
	Fear of stigma influencing behaviours.	"*…there is almost like a stigma against it like as soon as you say like healthy food people kinda go "aw it's not as good as like unhealthy… food or something*" (B4m)
		"*There's kind of a stigma that going to Weightwatchers, Scottish Slimmers, the NHS thing … is admitting you are wrong, kind of. So, it's like going to AA, Alcoholics Anonymous, you are admitting you've got a problem, whereas they are just trying to better themselves. "* (F2m)
	Negative consequences promoting healthier behaviour.	"*When I was growing up I was like, "I don't need to worry about that, that's not going to happen" but now I think I'm getting older and seeing friends who have problems with their health because they haven't exercised or because they've eaten badly. It made me think it's a lot more realistic so I pay a lot more attention now.*" (C3f)
**Perceptions and Attitudes Towards Health Messages**	Volume of healthy and unhealthy messages.	"*I see sometimes like advertisements from like gyms and stuff but like their, their just gyms. I guess they should advertise but.*" (B1f)
		"*…like unhealthy food adverts that people are bombarded by some would say so easily, so it's difficult to separate and choose…"*(B3m)
	Observation of messages - passively or actively seeking.	*"Tumbler, Pinterest. I quite often look up ways to increase my liquid, I know it sounds really sad, but I like trying to follow stuff like that so looking them up on Pinterest and they always have really interesting ways that make it more fun rather than if you sat with eight glasses of water.* "(C3f)
		"Just Google. I go through a couple because sometimes when you go on it's just a load of rubbish and you have to keep looking to find the right answer."(P1f)
	Perceptions of large corporations.	"*I think a lot of the companies like Coca Cola, and stuff like that, kids will drink that, or eat that without even thinking, just need to get more of that it's bad for you into their brains, and to cut down, or limit yourself to a certain amount every week."*(F1m)
		"it's like, "Oh boy, look, it's more fast food". It's like it's being shoved down your throat. It's like, "Come and eat here. Don't be healthy."(S1f)

		" I think that um if you've got an advertisement for healthy eating and an advertisement for a big multi-national, ya know, Coca-cola or whatever, you're obviously gonna recognise and see that one more often aren't you so. Yeah."(B2m)
**Facilitators and Barriers**	Consistent barriers to healthy choices.	"*It's like if you don't eat this, you're never going to do that. But it's not like that… it's not always about your five a day…I could eat a bag of bananas and it's not going to turn me into Superman ...Instead of going, "I've had five a day, now I can go and eat a bag of crisps", keep going, you don't just have to stop on the five a day*" (R1m)
		"...don't just say, "Eat this", say, "Okay, eat less of this" and swaps for practical actually achievable swaps, because if someone's eating half a pack of biscuits a day they're not just going to stop that and replace it with carrot sticks" (N1f)
		"*So, it's practicality over healthiness*." (F2m)
		"*I think there's also a thing of like not being bothered… sometimes like I can't be bothered cooking so I will just order something in and that is usually something quite unhealthy*."(B1f)
	Time constraints.	*"I think… they are a lot more work driven nowadays, like it's hard for them to go home and cook…. It's easy just to chuck something in the oven that is not healthy, rather than make something from scratch that is going to be healthy."* (F1m)
	Cost.	"*…I'm pretty heavily swayed by price, saying about how like an apple and like bananas are like expensive. I, I always think like their quite expensive*."(B2m)
		"I think definitely, recently cost is more of an issue, because people are wanting to stick to a weekly budget or whatever for food, and they go, "I don't have to do anything, I can go to McDonalds, and it's the cheapest thing I can get". (F2m)
		"*Because it's not cheap, being healthy. The food products, gym memberships, supplements, whatever and then if you're buying on top the books and the regimes and personal trainers, it's not easy for someone who has not got a lot of money. So, especially social media where everything is free and it's there at your fingers. It sounds quite ironic talking about health and saying just to go on your phone but it is a lot easier and quicker*." (R2m)
		" …You know you can either buy a 40p apple or you can buy like a bar of chocolate which is like you know… the price per weight is better… for chocolate than it is... Than like the fruit"(B2m)
	Goal setting.	*"It's not really important if you have a banana and an apple really when you're in that situation. But if it's vice versa, then it is. Or if you're at a weight that you're comfortable at or a lifestyle that you're comfortable at then it does give you general health and everything on top is health. But I think it's so broad that it doesn't apply. Everyone knows that and everyone is like, "I eat five a day" so no-one is inspired by that at all. Or drink this much water, or do that, it doesn't -- you never see anything that is like, "I ate my five a day and that's why I look like this", do you know what I mean?"* (R2m)
		"It's all very specific in books and posters and leaflets, it's usually aimed at people losing weight from fat to skinny.

		Whereas the internet there is such a wide variety of stuff, you can get anything and it is free…" (R2m)
		"Yeah definitely like most people who do it have specific goals that they want to reach" (B3m)
	Food appearance and taste.	*"…a McDonalds cheeseburger, is £1, whereas if you go and get a salad from somewhere it is £5. Why would you pay £5 for a salad, when you can get something lovely processed, cheesy and meaty for £1?* "(F2m)
		"*…you know it's going to work and that it's a nice meal. If they're saying, "This is really nice and I enjoyed it for my supper" then you know that you're going to like it. If you don't like tuna and you end up having tuna, you wouldn't be happy*" (P1f)
	Competition and challenges.	*"I just hate losing. If I play a sport and I lose, I will make sure the next time I am stronger, faster, whatever, so I don't lose."*(F2m)
		"We're all competitive as well."(C1m)
		"There are apps where you can track your activity with your friends." (C2f)
		"…I'm quite competitive so… it does make something a little bit better, even when it's like running apps, like I had… one which was good and it just told you how fast you're running and you know every 500 metres so you need to like up the pace or something. Even stuff like that makes you run like so much quicker"(B4m)
		"… It's like, "I'm a bit lower than my daily target with my friends" so next day you have that urge and motivation to… either show off or to prove to yourself that you can do it." (C1m)
**Improving the Usability of Messages**	Format of messages.	"*There's quite a few other ones that I see just people tagging me in, not tagging me in but they're sharing on their walls that I get to see. um other than that I don't really see many leaflets outside of doctors' offices or pharmacies or anything.*" (B2m)
		"*… And then Couch to 5K, you can almost make it a bit like a game because you get your stars after each run and you rate it"*(C3f)
		"*I think ways that are really good are little tips and advice that you see sometimes in leaflets just about little things like how to up your water.*" (C3f)
		"… I'm not going to read stuff but… it's just so much easier to watch a video… with all like the clicks like you get on Facebook it's like aw it's not a video even though its interesting" (B4m)
		"I'm I'm not a big fan of reading if I look at this I just see words... I just switch off" (B1f)
		"*The thing with social media is that you can do anything. You can put videos, interviews and stuff like that and it gets people a lot more involved and it's more inspiration than a bland bit of paper.*" (R2m)

		"*I like that they're quite clear, short. I like the… poster ones that show you… what could happen kind of thing. I like the visual ones more than probably the ones that are all words*."(C3f)
	Perceptions of current messages.	"*Yeah, like going back to even to the fact that we were saying there's always these kind of messages in doctors' surgeries but you know, a lot of young people well should hopefully be quite healthy and won't need to go to the doctor that often. So yeah, on things like social media that might be a platform that's more accessible for them and they're going to see more and you know, so those messages might have more of an effect."*(N3f)
		" Yeah well it's just like it's just a bit.... it's really dull. You don't even looking at it you kinda go like oh I'm going to stay hydrated... cause it says I need to drink water like... yeah."(B4m)
		"And ones like this one where it's almost just like saying it cause it has to say it cause its really well I'm not well it's kinda pathetic it's like oh stay hydrated, sleep... I dunno... eat slightly better."(B4m)
		"it's almost like quite demeaning… it's bringing you down to like a low level"(B2m)
	Improvements in presentation to be more memorable	*"…it needs to be more short and snappy and noticeable. You asked us earlier, what health messages we could pull off the top of our head. There is none of them, we can't think of anything, but if you asked us to recite the theme tune for the Hastings Direct TV ad, you'd be able to tell me the phone number! Because you'll sing the jingle in your head, and it's memorable…"*(F2m)
	Linking messages with a usable form.	*"I think it would it would have to include recipes as well… not just general information cause I wouldn't look at it*" (B1f)
	Using social media to promote health.	*"Sometimes on Facebook it comes up with things like that from the things you've Googled, so it's all linked, so if you haven't looked at anything on the NHS or searched anything healthy like eating or physical activity it might not come up."* (N2f)
		"*So I think for our kind of age demographic social media is probably going to be one of the most powerful methods of getting messages across."*(N3f)
	Reminders for behaviour.	"*Something that you can like a sly reminder, something that you haven't been on your run today…or the last week*" (B4m)
		"Yeah, every hour if you don't move 250 steps it vibrates so you've got to get up and walk so it's good" (P1f)

Supplementary Material 2

Transcript demonstrating categories identified.

BG3: It's like people create an identity for themselves it's like oh like I don't exercise, (BG4: hmm) and even if it's, like they don't understand that something as simple as going for a run every morning can help them in so many ways. And it's just like they just think like to exercise means you have to fit into this like rigorous (BG4: yeah) daily meeting limit like it's like not really as complicated as it seems.

BG4: Do you remember like, a few months ago when like the "Dad Bod" started kicking off (BG3: Hmm) and like within like I dunno within like male culture it was almost like aw actually now it's really good to have like, I'm not saying it's you know bad to have like a Dad Bod but you know like promoting an unhealthy way of living was actually seen as socially quite popular. Which is, yeah you know like, yeah, which is a bit strange like that it isn't like to say there is a happy medium like you have all those fitness gym freaks who ya know are like leaned and super toned (BG3: Yeah, yeah exactly) then you know you've got like the guys who just like goes out and gets drunk.

BG2: That's what like the body image thing I was thinking about earlier, um like I was saying Men's Health and stuff on Facebook. It's just full of these like hench dudes like just like lifting weights and stuff (BG3: Yeah, exactly, [inaudible comment follows at 11:24]). And I'm just like, its kinda unrealistic I feel (BG1 Interrupts: yeah you kinda have to be like hench before you start). I would say like I'm pretty healthy as I stand right now and I think I like am quite good at managing my diet but I always want to try and like make myself fitter just because I like the way that that looks. (BG3: Hmm). But I think that it's kinda unrealistic and that's maybe like why I, why I train, (BG3:yeah) yeah.

BG3: It's like they think that oh I'll never get to that so but they don't realise how hard it is like (BG2 +1: yeah) even if that person doesn't look like that all the time.

BG4: Especially when that, like that you've got between three to six percent body fat. (BG3: yeah, exactly). And that's like really unhealthy (BG3 +BG2: exactly, yeah) and you've can't maintain it. (Interruptions BG3: that's like worse than not having [trails off] BG2: exactly yeah, you can't maintain)

I1: yeah, that's really, really interesting, um, from what you've been saying it seems like a lot of the advertisements and messages you've guys seem to notice the most is ones that come from like private companies or like magazines or social media (BG2 + 1: uhmmm). Where as these sorts of messages don't seem to be out there as much, do you think that there's anything that you would change about these to try and get them more into sort of like young adults [trails off]

BG3: Well I think one of the good things that advertising agencies have done is use popular stars so around about the Olympics there was a lot of adverts with Jessica Ennis promoting, and Mo Farah promoting health and healthy eating (BG2: hmmm). Which was good, people were excited about them. So people were excited to see and you know like get into health and stuff. People were doing it with Anthony Joshua recently as well (BG4: I was going to say yeah) so that's quite like a nice a nice way to go oh look at this cool new person you could do this as well. So, I guess that's an effective way to do it.

Categories and key themes

1)Exposure to Health Messages over time

- Messages and influencing factors in childhood
- Schools- messages and experiences
- Messages available at College and roles in health
- Messages available from University
- Messages and behaviours within working environments
- Behaviours in the family home
- University lifestyle
- Knowledge of healthy living and behaviours

2) Chains of healthy or unhealthy behaviours

- Behaviour cycles
- Influencing factors for healthy behaviour – Alcohol consumption, social activities, stress, boredom
- Trust – figures of trust, role models, social norms, relatability
- Consequences of unhealthy choices
- Fear- stigmas, social media appearance

3) Perceptions and attitudes towards health messages

- Health messages --- passively available// actively looked for
- Volume of messages available ---- health messages// unhealthy messages
- Message awareness
- Perceptions --messages available// made on health// of Businesses and Large corporations
- Attitudes towards advertisements

4) Facilitators and barriers

- Time constraints
- Accessibility – health promotion materials, resources, healthy options
- Food taste and appearance
- Rigid messages
- Diversity for lifestyle choices
- Convenience factors
- Goal setting and targets
- Competition and setting challenges

- Reminders and notification for healthy behaviours
- Price of healthy living
- Personal experiences with weight management

5) Improving the usability of health messages

- Resources available- social media, TV, smart phones, leaflets, apps, fit-bits, books
- Message successes – visual, content. Interactive, personalisation
- Message appropriateness
- Message improvements
- Healthy recipes and information about healthy choices
